# Case 4 - A 67 Year-Old Man with Aortic Regurgitation Who Presented Syncope Followed by Shock

**DOI:** 10.5935/abc.20160125

**Published:** 2016-08

**Authors:** Desiderio Favarato, Luiz Alberto Benvenuti

**Affiliations:** Instituto do Coração (InCor) HC-FMUSP, São Paulo, SP - Brazil

**Keywords:** Aortic Valve Insufficiency, Atrial Fibrillation, Syncope, Shock, Cardiogenic

RFA, male, 67 years old, admitted for decreased level of consciousness and respiratory insufficiency (May 13^th^ 2011).

Patient under follow-up at InCor for a double aortic lesion since 2003. The patient had progressed asymptomatic in regards to dyspnea and chest pains. Many years ago, the patient underwent surgery for peptic ulcer.

He had presented with at least three episodes of hemiparesis with complete regression during follow up and, cognitive deficit, preventing him from correctly following medical prescriptions, occurred as a sequela. Atrial fibrillation was found during outpatient care. There was no use of oral anticoagulant due to the reported cognitive deficit.

During follow-up, the patient underwent several echocardiographic evaluations that showed dilatation and mild dysfunction of the left ventricle (Table 1).

**Table 1:** Weight, height and evolutionary echocardiographic data

	**2003**	**2009**	**2010**	**May 2011**	**May 2011**
Weight (kg)	75	58	56	60	65
Aorta (mm)	35	43	42	44	44
LA (mm)	43	47	46	39	39
RV (mm)	Normal	Normal	Normal	Normal	Normal
Septum (mm)	12	12	12	10	11
Posterior wall (mm)	13	12	10	9	10
LV (diast/syst) (mm)	70/56	65/50	68/41	50/-	62/52
LVEF (%)	48	45	69	40	35
Degree of aortic insufficiency	Severe	Severe	Severe	Moderate	Severe
Gradient LV- Ao (mmHg)			22	-	20
Ao sinuses (mm)			42	-	
- Sinotubular junction (mm)			33	-	
- Ascending (mm)			34	-	
- Arc (mm)			26	-	
Systolic pressure of the PT (mm Hg)			33	-	
Mitral Valve	Insuf	Insuf+	Insuf +	Insuf +	Insuf +
LV kinesis	Hypo	Diffuse hypo	Diffuse hypo +	Inferior and inferolateral and anterolateral akinesia	Inferior and inferolateral and anterolateral akinesia

LA: left atrium; LV: left ventricle; RV: right ventricle; EF: ejection fraction; Ao: aorta; PT: pulmonary trunk; diast/syst: diastolic/systolic diameterratio.

In November of 2010, during outpatient care, the patient complained of adynamia, weakness and weight loss in the previous six months and, about one month before the appointment, he had presented gastrointestinal bleeding, for which he needed a blood transfusion. On that occasion, his red blood cell count was 6 g/dL (Table 2). The patient was on acetylsalicylic acid 100 mg, enalapril 40 mg, digoxin 0.25 mg, furosemide 40 mg, potassium chloride 600 mg, ferrous sulfate 2x/day and amitriptyline 25 mg daily.

**Table 2:** Laboratory exams

	**2006**	**2009**	**2010**	**May 13^th^ 2011**	**May 15^th^ 2011**	**May 16^th^ 2011**
Erythrocytes: million/mm^3^	4.9	4.7	3.3	3.9	4.0	3.8
Hemoglobin (g/dL)	12.6	12.9	6.0	11.4	11.5	11.1
Leukocytes /mm^3^	7900	4400	3200	7460*	10280**	12890***
Platelets /mrr^3^	252000	206000	284000	77000	105000	94000
Potassium (mEq/L)	4.4	4.9	4.5	3.6		
Sodium (mEq/L)	140	136	137	152		
Cholesterol (mg/dL)	162	188	130			
HDL- C (mg/dL)	33	36	33			
LDL-C (mg/dL)	102	123	84			
Triglycerides (mg/dL)	133	146	66			
Glucose (mg/dL)		92	95			
TSH (µUI/mL)	0.848	0.952	0.562			
Free T4 (ng/dL)		2	0.95			
Creatinine (mg/dL) FG (ml/min/1.73 m^2^)	1.7	1.37 (55)	1.26 (> 60)	4.74 (13)	3.4 (18)	6.41 (9)
Urea (mg/dL)	55	42	50	247	147	218
Uric acid (mg/dL)		6.7	5.5			
CRP (mg/L)			2.09	272		312
Urine I						
Density			1.019			
Proteins (g/L)			0.29			
Leukocytes (/mL)			104000			
RBC (/mL)			16000			
CK MB (ng/mL)				32.8	13.0	
Troponine I (ng/mL)				> 100	> 100	
PT (INR)				1.4	1.40	2.6
APTT (rat.)				1.24	1.32	Incoagulable
Venous blood gas						
pH				7.29		7.11
pCO_2_ (mmHg)				48.4		47.6
pO_2_ (mm Hg)				48.4		46.6
O_2_ saturation (%)				72.4		83.2
Bicarbonate (mEq/L)				22.7		14.6
Base excess (mEq/L)				(-) 3.4		(-) 14.2

* 25% band; 63% segmented; 8% lymphocytes e 4% monocytes.

** 16% band; 81% segmented; 2% lymphocytes e 1% monocytes.

*** 91% neutrophils; 6% lymphocytes e 3% monocytes.

TSH: thyroid stimulating hormone; GF: glomerular filtration; CRP: C-reactive protein; CK MB: isoenzyme of creatine kinase; PT: pulmonary trunk; APTT: Activated Partial Thromboplastin Time.

ECG showed atrial fibrillation rhythm, left bundle branch block, and left ventricular overload.

Echocardiogram showed left ventricle dilatation and hypertrophy and severe aortic insufficiency (Table 1).

Upper digestive endoscopy revealed gastritis and anastomotic mouth ulcer from a Billroth II reconstruction gastrectomy surgery.

Chest X-Ray (Sep. 2010) showed free diaphragmatic domes and sinuses, lung parenchyma with normal transparency. Normal pulmonary vascularization, hilar configuration, topography and dimensions. The aorta was elongated with wall calcification and presence of cardiomegaly with normal mean arch.

The patient remained on follow-up and a new upper digestive endoscopy was prescribed. The endoscopy was done on March of 2011 and showed partial Billroth II reconstruction gastrectomy and enanthematic gastritis of the stump. Biopsy showed moderate chronic active gastritis with regenerative foveolar hyperplasia, lymphoid aggregates, and complete intestinal metaplasia in the pyloric mucosa. *Helicobacter pylori* culture was negative.

In May of 2011, the patient was brought to the Emergency Room of the Hospital with syncopal episode followed by a decrease of consciousness level and dyspnea six days prior to admission.

Physical examination (at 20 h and 45 m on May 12^th^ 2011) revealed decreased level of consciousness, dehydration, uremic breath, and axillary temperature of 37.8°C. Heart rate was 121 bpm, blood pressure 135 x 66 mmHg. The patient required orotracheal intubation for respiratory support, and 500 mL of 0.9% sodium chloride was administered.

ECG (May 13^th^ 2011) showed atrial fibrillation rhythm with mean heart rate of 124 bpm, QRS duration of 108 ms, corrected QT duration of 437 ms, ÂQRS (-)60°, left bundle branch block, left ventricle overload, ventricular repolarization alterations with ST depression and inverted T waves in V_4_ to V_6_. (Figure 1).

**Figure 1:**
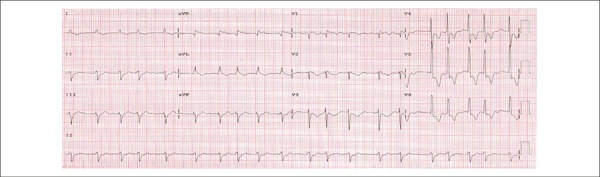
ECG. Atrial fibrillation rhythm, left bundle branch block.

Chest X-Ray (May 12^th^ 2013) showed cardiomegaly and pulmonary congestion (Figure 2).

**Figure 2:**
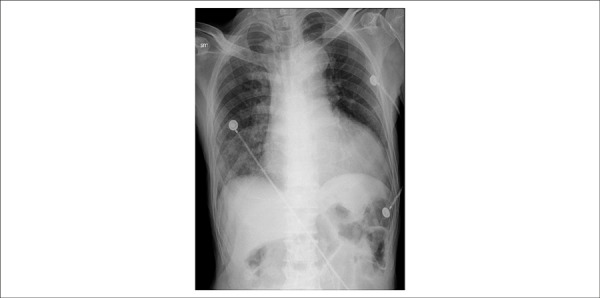
Chest X-Ray. Cardiomegaly and pulmonary congestion.

Labs (May 13^th^ 2011) revealed white blood cell count with a left shift, without leukocytosis; thrombocytopenia, renal insufficiency with uremia, mixed acidosis, and elevated myocardial injury markers, troponin I and CK-MB (Table 2).

Echocardiogram (May 13^th^ 2011) showed left ventricle with eccentric hypertrophy and decreased systolic function due to akinesia in the basal segments of the inferior and inferolateral walls and in the basal and mean segments of the anterolateral wall, as well as moderate aortic insufficiency (Table 1).

Head CT (May 13^th^ 2011) revealed attenuation of nonspecific periventricular white matter; right occipital hypoattenuating area, with right lateral ventricle dilatation, hypoattenuating areas in semi-oval center bilaterally.

Blood cultures collected on May 13^th^ 2011 were positive for methicillin sensitive *Staphylococcus epidermidis* and coagulase-negative *Staphylococcus (Staphylococcus lugdunensis).* Urine culture was positive for *Proteus mirabilis* and in the tracheal secretion there was growth of one million colonies of *Escherichia coli, Klebsiella pneumoniae* and *Staphylococcus aureus.* Initially, ceftraixona, clarithromycin and oxacillin were administered, and were later substituted for imipenem, vancomycin and an association of piperacillin and tazobactam.

After the first day of hospital admission, the patient progressed with hypotension and required the use of noradrenaline. He underwent dialysis for four hours on May 14^th^ 2011, presenting with hypotension in the last hour.

The patient presented an episode of hypoglycemia (22 mg/dL) and hypotension, which was reverted with intravenous glucose. Another complication was a right pneumothorax, which was drained (May 16^th^).

The patient maintained a decreased level of consciousness, even with no sedation, persistent fever and hemodynamic worsening, impeding hemodialysis on May 15^th^.

Lab exams revealed worsening of kidney function and acidemia (Table 2).

Transesophageal echocardiogram (May 15^th^) was not indicative of infective endocarditis.

A new chest X-Ray (May 15^th^) showed condensation of apical segments of the lower lobe (Figure 3). X-Ray of the abdomen revealed urinary retention.

**Figure 3:**
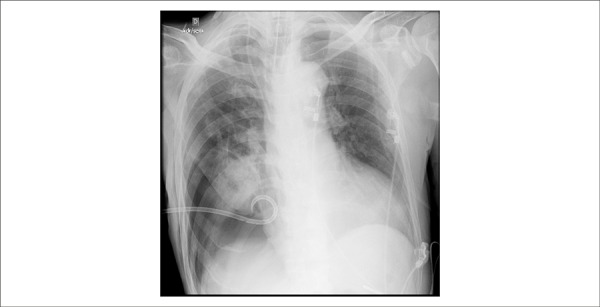
Chest X-Ray. Cardiomegaly and pulmonary condensation in the right inferior lobe.

Blood cultures collected on May 16^th^ 2011 were negative.

Presented asystolic cardiorespiratory arrest and died (May 16th 2011).

## Clinical aspects

Patient with aortic insufficiency diagnosed at 57 years of age, presenting with several transient ischemic attacks simultaneous to atrial fibrillation. Presented with upper digestive bleeding and, finally, syncope followed by decreased consciousness and complex mixed shock with acute renal failure.

The patient showed normal aortic insufficiency clinical evolution, that is, progressive dilatation of the left ventricle with few cardiovascular symptoms. Generally, aortic insufficiency progression is slow and the left ventricle has time to dilate and handle high regurgitating volumes with normal filling pressure. This good compliance maintenance and low end-diastolic pressure has little repercussion on the left atrium and on the pulmonary venocapillary territory, thus there is no increase in liquid content in the lungs and dyspnea appears as a late symptom.

With the advancement and progression of myocardial fibrosis and other myocardial mechanic alterations, ventricular dysfunction occurs and symptoms as dyspnea, heart failure and, more rarely, angina, appear.^1^

Due to a lack of symptoms, the best moment for the recommendation of surgery is widely discussed. In a series of 246 patients at Mayo Clinic, followed for 10 years, there was an occurrence of cardiovascular events in 83%, surgical repair in 62%, heart failure in 47%, and vascular complications in 15%. The functional class of heart failure greatly influenced 10-year survival of patients - 75% for those in Class I NYHA, 59% for those in Class II, and 28% for those in Classes III and IV. Among echocardiographic indices, end-systolic volume corrected for body surface was a good prognosis indicator: 81% survival for patients with systolic volume under 25 L/m^2^, and 34% for those with systolic volume of 25 L/m^2^ or more. Ejection fraction was also a mortality indicator: for those with an ejection fraction under 50%, there was 74% mortality; and for patients with an ejection fraction over 50%, there was 35% mortality.^2^

Another widely used index is the left ventricle systolic diameter with cut-off value of 55 mm, because, when it is higher, even with valve replacement surgery, survival is approximately 30% in five years. However, when the cut-off value is under 55 mm, survival is around 90%. The use of these indices determines a symptom-independent prognosis.^3-5^

The most likely prognosis of the valve disease does not seem to be rheumatic disease, because the diagnosis was very late and there was no previous history of rheumatic fever. It also does not seem to be related to Marfan's syndrome because of the patient's age and lack of involvement of the aorta and the mitral valve, with no valve prolapse in the latter.

The appearance of atrial fibrillation may contribute to the decompensation of heart failure, because atrial contraction may be responsible for up to 30% of the systolic volume in senior patients, patients with myocardial hypertrophy, or ventricular dysfunction, all of whom present with reduced ventricular compliance and decreased protodiastolic filling.^6,7^

Ejection fraction and tolerance to effort greatly decrease with the appearance of atrial fibrillation, even in patients without valvulopathy.^8^

In regards to syncope followed by low cardiac output presented by the patient, they may be secondary to some events that notoriously decompensate individuals with cardiac dysfunction, such as pulmonary thromboembolism, bradyarrhythmias or tachyarrhythmias, or even acute ischemic syndrome with malignant arrhythmias.

The occurrence of syncope in aortic valve disease is more common in aortic stenosis, which, along with angina and dyspnea, combine the classic symptoms of this disease.^9^ In aortic stenosis, it occurs more frequently during effort; however, it can occur at rest if there is intermittent total atrioventricular block. Nevertheless, syncope is not a common symptom of aortic insufficiency, except for acute aortic insufficiency secondary to ascending aortic dissection, which does not appear to have occurred in this current case.^10^

Massive pulmonary thromboembolism may present with syncope and shock. However, echocardiograms did not show right ventricular dysfunction or dilatation as would be expected in this case.^11^

Acute coronary condition was favored by the appearance of inferior, inferolateral and anterolateral wall akinesia in the echocardiograms during the last hospital admission and by the significant increase of troponin, even though there was no major increase of CK-MB or Q waves in the electrocardiogram.

Duke criteria for diagnosing infectious endocarditis are divided into bigger criteria (blood culture with typical germ and echocardiographic findings of vegetation or perivalvular abscess) and six smaller criteria (predisposition, fever, vascular phenomena, indicative echocardiogram, and suggestive microbiological findings).^12^

Endocarditis cannot be ruled out because the patient, who had valvulopathy, presented with fever, worsened hemodynamics and growth of Gram positive cocci in the blood culture. However, no worsening of aortic regurgitation degree was detected and there was no presence of vegetations on heart valves in the transesophageal echocardiogram.

Mansur et al.^13^ showed that the best predictors of hospital death in cases of endocarditis were previous cardiac state, causative microorganism, complication occurrence, and white blood cell count.

Pneumonic condition was probably of bacterium-origin and may have aggravated the patient's condition. (**Dr. Desiderio Favarato**)

**Differential diagnoses:** Aortic insufficiency of undetermined etiology, ischemic heart disease with acute myocardial infarction and sepsis of pulmonary origin. (**Dr. Desiderio Favarato**)

## Necropsy

**Figure 4:**
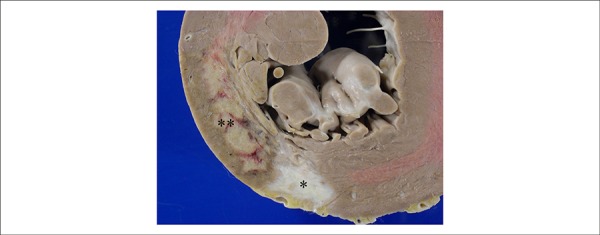
Cross section of the left ventricle showing area of healed infarction, with fibrous replacement of the myocardium on the posterior wall (asterisk) and recent transmural infarction on the posterolateral wall (double asterisk).

**Figure 5:**
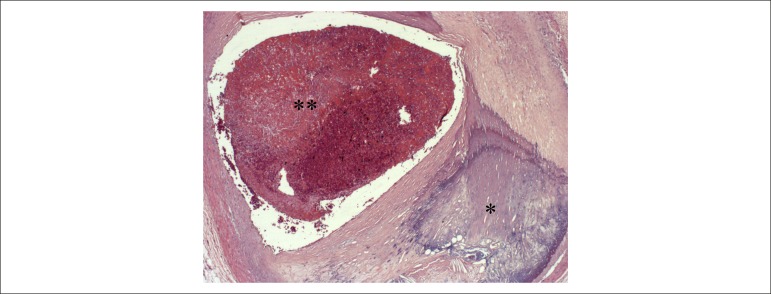
Histological section of the initial segment of the left marginal artery showing calcified atherosclerotic fibrous plaque (asterisk) and recent luminal thrombosis (double asterisk). Hematoxylin-eosin, X 15.

**Figure 6:**
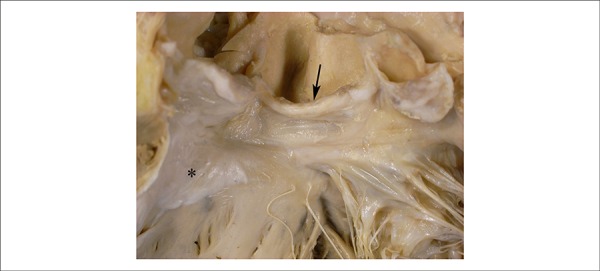
Trivalve aortic valve with collapsed semilunar, showing retraction and thickening of the free edge (arrow). Note the milky focal thickening of the endocardium of the left ventricular outflow tract (asterisk), secondary to regurgitant jet of aortic insufficiency.

**Figure 7:**
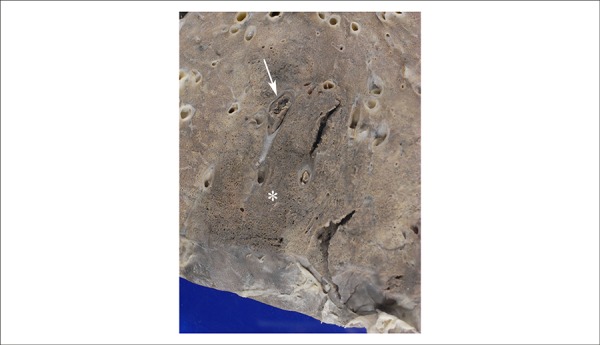
Microscopic detail of the right lung inferior lobe showing arterial occlusion by thromboembolus (arrow) and area of recent hemorrhagic infarction (asterisk).

**Figure 8:**
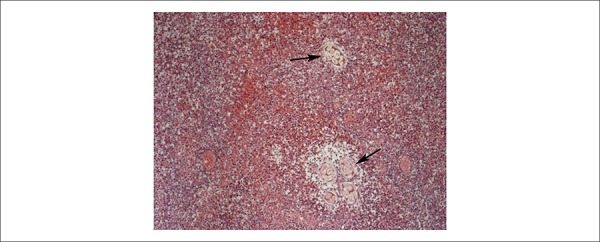
Histological section of the lung showing bronchopneumonia with presence of remnants of vegetable cells (arrows), characterizing etiology of aspiration. Hematoxylin-eosin, X 50.

The heart weighed 684 g. The left ventricle showed severe hypertrophy with mild dilation of the cavity, focal area of fibrous myocardial replacement of the posterior wall (infarction scar) and recent transmural infarction of the posterolateral wall (Figure 4). The atrial septum was intact and the foramen ovale was closed. Histologic exam confirmed acute infarction with histological dating compatible to 2-5 days of evolution. Coronary arteries presented complex atherosclerosis, with areas of calcification; there was 80% obstruction of the distal segment of the right coronary, and 60% of the initial segment of the anterior interventricular artery. Circumflex and left marginal arteries presented occlusive recent thrombosis, as well as atherosclerotic plaque (Figure 5). Aortic valve was trivulvular, with collapsed semilunars, showing fibrous retraction of the free rim and calcification points. There were no thrombi, vegetations or infectious endocarditis (Figure 6). In the lungs, we detected recent, bilateral, thromboemboli, with areas of recent hemorrhagic infarction in the superior and right-inferior lobes (Figure 7). There was bronchopneumonia in the inferior lobe of the right lung, along with fibrinopurulent pleuritis, with presence of Gram positive cocci colonies and food debris in alveolar spaces, characterizing aspiration etiology (Figure 8)

The encephalon presented old infarction area, healed and cavitated, in the right occipital pole. There was partial gastrectomy, with Billroth II reconstruction, with no abnormalities. We found horseshoe kidney and the aorta presented complex atherosclerosis, with calcified fibrofatty plaques. (**Dr. Luiz Alberto Benvenuti**)

**Anatomopathological diagnosis -** Atherosclerotic ischemic heart disease and acute myocardial infarction; degenerative aortic valve disease, with functional insufficiency; thromboembolism and recent pulmonary infarction; aspiration bronchopneumonia; previous partial gastrectomy; horseshoe kidney. (**Dr. Luiz Alberto Benvenuti**)

## Comments

Case of 67 year-old man, on follow up for many years at InCor for aortic valve disease with insufficiency. Presented with a history of partial gastrectomy and previous strokes, with cognitive deficit. In the period of outpatient monitoring, the patient presented episodes of digestive bleeding, adynamia, progressive weakness and weight loss. Digestive biopsy revealed chronic gastritis with no evidence of neoplasia. After admission to the ER with a history of syncope followed by decreased level of consciousness and dyspnea, the patient remained in the hospital for three days, having died at the end of the period. During his hospital stay, acute myocardial infarction and probable infection with non-defined focus were diagnosed.

Necropsy confirmed the presence of acute myocardial infarction on the posterolateral wall of the left ventricle. The infarction was based on the recent thrombotic occlusion of the circumflex and left marginal coronary arteries, previously affected by atherosclerotic disease - main cause of ischemic heart disease.^14^ The patient also had bilateral pulmonary thromboembolism with presence of recent hemorrhagic infarction areas in the right lung. Interestingly, the simultaneous occurrence of acute myocardial infarction and pulmonary thromboembolism is unusual, leading to diagnostic difficulties since both conditions present with similar signs and symptoms.^15^ Necropsy confirmed the presence of chronic aortic valve disease with insufficiency, of degenerative etiology. There was no infectious endocarditis, consistently with the clinical diagnosis. On the other hand, we confirmed the presence of infectious disease, also clinically suspected, established in the right lung as aspiration bronchopneumonia. Such infection, probably pre-existent, was related to the previous history of syncope and decreased level of consciousness, because the patient already presented infectious evidence at admission. There was no neoplastic lesion in the gastrectomy stump and horseshoe kidney was found during necropsy. Death was caused by mixed hemodynamic cardiogenic infectious shock. (**Dr. Luiz Alberto Benvenuti**)

**Section editor:** Alfredo José Mansur (ajmansur@incor.usp.br)

**Associated editors:** Desidério Favarato (dclfavarato@incor.usp.br), Vera Demarchi Aiello (anpvera@incor.usp.br)
